# The problematic space between art, ambition, and gameplay: *The Medium *and the issues concerning difficult subject matter and gameplay in games

**DOI:** 10.12688/f1000research.133472.2

**Published:** 2024-01-05

**Authors:** Katarzyna Marak, Miłosz Markocki, Krzysztof Chmielewski, Dariusz Brzostek

**Affiliations:** 1 Uniwersytet Mikolaja Kopernika w Toruniu, Torun, Kuyavian-Pomeranian Voivodeship, 87-100, Poland; 2 Uniwersytet Kazimierza Wielkiego w Bydgoszczy, Bydgoszcz, Kuyavian-Pomeranian Voivodeship, 85-064, Poland

**Keywords:** digital game, art, The Medium, eudaimonic gratification, gameplay, ludonarrative dissonance

## Abstract

This article identifies and examines a couple of selected issues regarding speculative digital games which endeavor to deal with serious subject matter. Due to the fact that speculative games are exceptionally well suited for symbolic representation of sensitive topics, they allow their creators to conceive ambitious projects that aspire to be great works of audiovisual art. However, because game texts belong to a very specific medium, it is not always possible to realize this ambition in the context of gameplay. For reasons of space and focus this article uses one particular game, *The Medium* (2021), to serve as the primary example of how the problems which occur in the process of combining specific, engaging gameplay with serious, sensitive subject matter, lead to situations in which a game can fail to fulfill the player’s expectations. By analyzing the structure, gameplay, and storytelling tools employed in *The Medium*, the article places emphasis on the significance of the possible tensions between the intention of the designers of the game experience and the experiences, ideas, and interpretations the players themselves bring to the game text.

## Introduction

As games become ever more intricate, moving, and challenging, game studies have been moving away from looking at game texts as mere sequences of scripted choices contained in designed spaces; instead, in addition to examining the ludic profile of game texts, many analyses now tend to draw attention to “ideas of autonomy and control from both the player and designer perspectives” ( Rothschild *et al.* 2013: 83) as well. This article adopts the position that examining a game text from a perspective that takes into consideration the story-related, agency-related, and design-related elements of that text allows for a more comprehensive and productive study of games.

Of particular interest for this paper are the characteristics of fantasy, horror, and adventure games which aspire to portray, express, and convey ambitious themes, experiences, and stories; to this end, these game texts oftentimes include or rely on speculative elements, worlds, and scenarios, which allows them to move away from imitating reality, as well as the burden of the premise that “reality is objective and unambiguous” ( Oziewicz 2017). For this reason, such games tend to be exceptional vehicles for symbolic representation of sensitive topics and excel at presenting allegorical figures and scenarios referring to trauma. There are numerous titles whose goal it is to present serious subject matter as a unique immersive experience. Non-mimetic games can concern diverse sensitive topics, including obsession, guilt, penance, impotence, bigotry, or dejection, as can be seen in games such as: *Layers of Fear* (2016), *Hellblade: Senua’s Sacrifice* (2017), *Someday You’ll Return* (2020), *Silent Hill 2* (2001), *Silent Hill 3* (2003), *SOMA* (2015), *The Dark Pictures Anthology: House of Ashes* (2021), or *Scorn* (2022). These are, of course, only a few to name in the context of a larger body of games employing supernatural of scientific speculative concepts or scenarios in order to offer “occasions to reflect on our condition as human beings and to reflect on what makes our life meaningful” ( Cova *et al.* 2017: 349); the motivation to seek out narratives which elicit negative affective reactions (cf. Oliver & Bartsch 2010), i.e. eudaimonic motivations, leads the players to engage with texts which are able to provide them with satisfaction related to cognitive and emotional effort and experiences as they play the game: eudaimonic gratification.

Speculative games have at their disposal a whole range of reliable and effective cinematic and ludic tools. The employment of both makes it possible for the creators to include meaningful cultural details in their depicted worlds and build an atmosphere, as well as explore themes through visual design and *mise-en-scène.* The camera shots and shot composition help accentuate the most significant story elements, influencing the overall gameplay experience, similarly to game spaces, which contribute to the narrative and present challenges to the player. The complex, changeable nature of game characters, who can serve a function of a non-playable character, a protagonist, or an avatar, or even two of those at the same time, on the other hand, is inherent to the facilitation of ambiguity within a story framework.

This article aims at calling attention to the problems which occur and can be observed in some speculative games that attempt to combine specific, engaging gameplay with serious, sensitive subject matter, but are unsuccessful in doing so. To this end, this paper presents a case study of *The Medium*—a psychological horror video game developed and published by Bloober Team on January 28, 2021—and the ways in which it fails to fulfill the expectations of the player in the context of delivering eudaimonic gratification in being a game text, and not by reason of being a game text.

## Methods

This paper utilizes the method of case study as it is applied in cultural studies—an examination of “a typical cultural artifact and medium of modern culture, and [how] through studying its ‘story’or ‘biography’ one can learn a great deal about the ways in which culture works” ( Du Gay *et al.* 2013: xxix). In the case of *The Medium*, this approach constitutes qualitative research of the particular type of digital game texts and the ludonarrative dissonance typical of those texts. Even if a case study will not provide any general conclusions regarding the dissonance and balance between the ludic and the narrative, it still can name at least some constitutive principles observable in a particular body of game texts.

The primary focus of this text concerns game texts featuring narratives revolving around sensitive topics and themes. Of particular interest to our research and observations is the interplay between the face-value narratives produced by such games as well as their symbolic vocabulary. Even more importantly, the games we consider are ambitious both in terms of concept and narrative at high aesthetic level. They touch upon a range of topics from child abuse, mental illness, to seeking and finding closure, or striving to reflect affective, social, and political realities in individualized, allegorical manner. Apart from the titles mentioned above, there are many more game texts of that type, including *Among the Sleep* (2014), *The Beast Inside* (2019), *Blair Witch* (2019), *Detention* (2017), *The Dark Pictures Anthology: Little Hope* (2020), *Fran Bow* (2015), *Observer* (2017), *Perception* (2017), *Through The Woods* (2016), *Tell Me Why* (2020). Some of these games fulfill their task of engaging the player in their story as much as in gameplay ( *Layers of Fear*, *Hellblade: Senua’s Sacrifice*, *Detention*, or *SOMA*), while others fail in one or both of these goals ( *Someday You’ll Return*, *The Beast Inside*, or *Blair Witch*).

The game *The Medium* was selected as a representative example of a story-driven speculative game dealing with serious subject matter, which tells dramatic and intriguing story, but offers the players rather awkward gameplay. The case study method is supplemented by data obtained from Steam platform. The players’ reviews on Steam were reviewed manually on Mar 6-10, 2023, in 3 different categories provided by Steam (positive, negative, mixed); no automatized tool was used. A total of 5,292 reviews (all Steam reviews for *The Medium* available on Mar 6, 2023) was read in whole in search for contents relevant to the article, with the utmost focus on the gameplay and mechanics. Additionally, the close reading has been complemented with data gathered from letsplays of the game examined in the process of researching this article. The inclusion of diverse recordings of the performative experience of playing the game has allowed for the inclusion and exploration of the personal gameplay experience of other players and, therefore, a more comprehensive analysis ( Marak 2021). The purpose of the resulting analysis is the identification and overview of problems stemming from the necessity of translating a complex story into a satisfying gameplay experience. *The Medium* as a case study makes it possible to name the key issues resulting from the various restrictions placed on the player’s agency in relation to the narrative potential of the story, which are also present in some of the other similar story-driven speculative games.

### Ethical consideration

Ethical approval and consent were not required for this study as the study is of low risk, with no intervention, no correspondence with the Steam users involved, and the use of anonymised data.

### The story and characters

The events of *The Medium* take place in Poland. The story revolves around a young woman, Marianne, who is the player character and, seemingly, the center of the entire game narrative. Marianne happens to be a spirit medium, able to access the realm referred to as Spirit World, and capable of helping lost souls find rest. Alone and deprived of purpose after the death of her legal guardian, Jack Orkan, she is caught off guard by a phone call from a man who introduces himself as Thomas, and who claims to both understand her power and be able to explain its origin, as well as the nature of her recurring nightmares—on the condition Marianne meets him in person in the abandoned secluded Niwa Workers’ Resort. Upon her arrival, Marianne finds the building empty and in ruin, but full of traces, clues, and trapped souls connected to the appalling massacre that led to the closure of Niwa many years earlier. There is also another presence in Niwa, which Marianne later discovers to be the greatest threat in that place.

The two most noteworthy components of *The Medium,* informing its storytelling and its gameplay mechanics, are the concepts of splitting and superimposition, both involving the past and the present as well as the material and the spiritual. The depicted world does not merely include spirits and supernatural occurrences, but is in fact founded on the premise of reality consisting of two aspects meant to complement one another: the material world and the Spirit World. As a speculative game text, *The Medium* uses this idea to challenge “the materialist complacency that nothing exists beyond the phenomenal world” and expands its fictional reality beyond that which is tangible and explainable ( Oziewicz 2017), offering the player emotional experiences, encounters, and knowledge that otherwise would be inaccessible to them. Marianne’s power to access the Spirit Word makes her quite literally the focal point of the story and the gameplay experience. The game emphasizes the juxtaposition and superimposition of the past and the present by means of splitting and switching the virtual environment, which affects the gameplay to a great extent; the story, however, uses this premise to a slightly different effect.

In the context of the overall narrative, the focus on the past seems to portray Marianne as a character as someone outside of her own timeline. In some ways, Marianne appears to exist outside of the present. Although the game itself opens with narration delivered by Marianne, concerning later events, the gameplay begins in Jack’s apartment, who at this point in time is already dead. His business, a funeral home, is also related to handling the dead, and is depicted as having become irrelevant after his death, as Marianne makes no mention of keeping it. Financial future is not the only thing Marianne fails to refer to. The player never learns of any friends, interests, present obligations or future plans she might have. At this point, there is also no mention of any other family members aside from Jack. It is only later in the game that Marianne, and therefore the player, finds out about her biological father, Thomas Rekowicz, and elder sister, Lilianne. Even more importantly, Marianne’s power is subsequently explained to be a family trait—it is no longer treated in the story as a bizarre ability setting her apart from others, but as a gift inherited from her father. However, none of this information she actually receives from living people with whom she could engage in conversation.

The story of Marianne’s family is revealed gradually as the game progresses through visions of the past, encounters with spirit remains of people either dead or gone, or through retrospective sequences revolving around those people. Once in Niwa, Marianne meets Sadness, a child spirit who is afraid but refuses her help. More importantly, Sadness mentions her connection to the monster roaming the ruins of Niwa, which occasionally pursues Marianne obsessively—the Maw. The player, watching Marianne’s visions eventually discovers that Sadness is in fact the child spirit of Lilianne, who came into existence after Lilianne was sexually abused by their father’s trusted friend, Richard Tarkowski. Marianne later realizes that she is Lilianne’s younger sister, whom Thomas Rekowicz sent away for her own safety after a fire that nearly killed both of them. Marianne’s visions also show more of Niwa’s past, which allows the player to understand that Thomas Rekowicz was a medium similar to Marianne; his special ability consisted in trapping a person’s mind in their body. He was hiding at Niwa, in Poland, with his family, when he was found and attacked by Henry Wilk, a Security Service agent. Although Thomas Rekowicz was able to neutralize Henry, the fire set by the latter constitutes the most grievous and catastrophic event in the entire story of the game, since it is at that moment when young Lilianne, terrified and hurting, is tricked by the growing darkness inside her. In exchange for saving her and her baby sister’s—Marianne’s—life, Lilianne sets the darkness which has been growing in her ever since Richard’s assault free into the world, where it takes the monstrous form of the Maw, which the player can recognize as the monstrosity haunting Niwa’s grounds, and the fiend responsible for the massacre that led to the resort’s closure. Marianne also learns that her father, having lost his connection to the Spirit World during his confrontation with Henry, devoted the rest of his life to confine the Maw by creating a special, magically reinforced bunker in which Lilianne spent the majority of her life. None of this knowledge is optional, as the information is not embedded in the world in form of narrative elements such as letters or journals, or expressed in the game environment; instead, it is scripted as cut-scenes, which turn the player from an actor into a captive spectator (cf. Calleja 2011). As a result, Marianne, together with the player, is suddenly burdened with both the knowledge of, and the emotional responsibility to be invested in, events she was (apart from the night of the fire) never a part of, and family members she had never met (in a meaningful way she would be aware of). She therefore appears to have no place she could occupy in the timeline of that story, unlike, for example, Heather Mason in *Silent Hill 3*, who also learns a great deal about her past self throughout the game in order to understand her role in her present. A related issue is Marianne’s lack of apparent dramatic need in the context of the fact that *The Medium* is regarded as a text of the horror genre. To make another comparison, Senua in *Hellblade: Senua’s Sacrifice* sets out to face Hela driven by a sense of guilt and the hope of Dillion’s resurrection; in *Silent Hill 2* James Sunderland travels to Silent Hill in search for his wife, while the Artist in *Layers of Fear* rambles around his house because of the mad compulsion to complete the Painting. In contrast to the aforementioned characters, Marianne’s only motivation to go to Niwa is just a mysterious phone call made by a person claiming to be able to explain her nightmares. The dramatic need in horror games tends to require even greater suspension of disbelief than in other speculative games. However, the reason for Marianne to actually go to Niwa and stay there even after she realizes that the place is terribly dangerous is rather vague and inadequate; in consequence, affective identification with Marianne can become difficult, as she loses some credibility as a believable horror protagonist, and, as a result, she functions more as a blank canvas to encourage the players to focus more on the environmental storytelling rather than character-centric exploration.

In the context of *The Medium* as a narrative, there are far-reaching consequences of Marianne’s role and character arc within the framework of the game’s storytelling. Her dramatic need as a character becomes relevant only after the player is subjected to complete exposition through the sequences in which Marianne receives all the necessary visions, which means that each story fragment falls into place, leaving almost no potential questions or doubts. Thus, there are no Ingarden’s spots of indeterminacy or Iser’s textual gaps or blanks that would appear through the interaction with the player and need filling ( Sandvoss 2007: 28). Since the game story advances itself (cf. Bateman 2021), there is little effort required from the player to ponder and create meaning along the way. This means that *The Medium* as a narrative does not allow for any ambiguity in its story; this is a very important point, as ambiguity–as characterized by Cole and colleagues–tends to play

a major role in the emotional impact of the [independent story-based] games … By leaving space for the player to think and contemplate—unburdened by the requirements of completing functional challenges, the player is better able to emotionally invest, and subsequently receive a greater emotional return, in the diegesis ( Cole *et al.* 2015),

with the core pleasure offered to the player being “the resolution of tension within the narrative, emotional exploration of ambiguities within the diegesis, or identification with characters” ( Cole *et al.* 2015). Within the narrative of *The Medium*, there are few moments or places of ambiguity, apart from the very ending, which unfortunately in itself makes the resolution of tension impossible. Another aspect of the game which could potentially allow for ambiguity—but evidently does not—is the exceptional status of Spirit Marianne, or, in other words, Marianne’s Spirit Self. The Spirit Selves that the player encounters in the game are human spirits and monsters, which differ from and do not occupy the same space as the material selves of the humans they are parts of. Human spirits represent the given character as they were in the moment of their death, such as Spirit Jack, or in the moment of a severely traumatic experience. Sadness, the child spirit of Lilianne, is the most important among such characters, although the player is also introduced to the child spirits of Richard and Henry. Due to the fact that *The Medium* is a horror narrative, the monsters are the most visually striking Spirit Selves; the main antagonist, the Maw, is a humanoid creature born from Lilianne’s trauma, who indiscriminately destroys everything and kills anyone it can reach in both the material world and the Spirit World. The monstrous Spirit Self of Richard, meanwhile, known as the Childeater, is the result of Richard’s twisted obsession with little girls, which manifests itself in the form of a gargantuan lecherous, vaguely human-shaped entity with multiple arms and tentacles, which is a very direct manifestation of Richard’s tendencies; it does not seem to be able to affect the material world directly, only Richard’s behavior. Interestingly, in contrast to Sadness and Richard’s child Spirit Selves, Henry’s child Spirit Self seems to be the only child spirit not terrified of its monstrous Spirit Self. Instead, the child Spirit Self of Henry appears to be an extension of the Hound, a predatory monster living in Henry Wilk’s warped mind potentially since his childhood, whose canine shape crowned with an oversized human skull evidently points to associations with hunting, implying that the Hound may be always in control of adult Henry.

Only Marianne’s Spirit Self is the same in identity, appearance and personality as her material self. Therefore, Spirit Marianne is one of a kind, unlike any other character portrayed in the game as she is merely Marianne’s reflection in the Spirit World. She never speaks and she never does anything Marianne does not do in the material world; even in the sequences when Marianne needs to leave her physical body in order to find or access something in the Spirit World, her Spirit Self is indistinguishable from material Marianne as an avatar. She literally occupies the same space and time. This is an informed choice on the part of the creators, which makes sense from the point of view of actual gameplay, but it is important to note that other options were available. For example, it is possible to have two individual avatars at the center of the game narrative, as can be seen in *Tell Me Why* (2020), where the player switches between the twins who are the protagonists of the story. Similarly, it is possible to have a protagonist who is materially confined in specific locations of the game, and a separate playable character who exists on a different plane and is therefore unhindered by material obstacles, as can be seen respectively with Jodie and Aiden in *Beyond: Two Souls* (2013). The significance of the choice made by the creators of *The Medium* is best seen in the last cut-scene of the game, where a very different type of split screen is employed, and Marianne is never shown as two separate characters. Accordingly, Marianne’s Spirit Self is completely undeveloped as a character, in unlike Spirit Thomas, who is the Spirit Self of Thomas Rekowicz. Spirit Thomas is clearly an independent entity—ruthless, sarcastic, and more assertive than Thomas himself, bearing symbolic marks reflecting Thomas Rekowicz’s traumatic and violent life. In contrast, Spirit Marianne is not her own person–apart from the burns Marianne suffered in the fire as a baby, which in the Spirit World are expressed visually as fungal-like growth on her burned shoulder, there are no other indicators of her life experiences, as if Spirit Marianne barely existed outside of the context of the fire.

This also means that Marianne is the only character who never engages in any sort of internal dialogue with her own Spirit Self. Consequently, the player is unable to gain any additional insight into Marianne’s personality, feelings, or thoughts. What is more, Marianne’s death would be final, since the game earlier establishes that Marianne’s Spirit Self cannot survive for longer periods of time away from Marianne’s material body. As such, the character of Spirit Marianne is primarily a mere placeholder for material Marianne, being removed from the structure of character building within the game; taking into consideration the fact that having an independent Spirit Self is a dominant element of character building in *The Medium*, Marianne without an independent Spirit Self becomes the exception that proves the rule, making her even more unique. However, this uniqueness limits the potential for ambiguity in *The Medium* even further.

### The space and events

Stories in games are more than simply sequences of events, as their narratives emerge from game spaces, which sometimes incorporate elements of designed experiences ( Rothschild *et al.* 2013: 83). Spacial mechanics of the narrative elements play an important part in shaping the gameful experience, as well as the player’s subjective appreciation of the game’s storytelling. It is narrative elements that make the game space meaningful, while the space itself allows for a specific arrangement of those elements, and the last and “most important element that needs positioning is the player” (Nitsche 2008: 44-45). It is the player who makes sense of the in-game events and situations, but the process itself—especially in speculative games—tends to be “evoked and directed by evocative narrative elements, formed by encounters or situations in the game that prime some form of comprehension” (Nitsche 2008: 44). The space of the game plays the central role in the meaning-making process, due to the fact that the player’s participation and their comprehension of the game world take place in a navigable, structured virtual space (Nitsche 2008: 159). The space in the game is both contextualized and motivated by the game’s fictional setting, and includes a variety of elements from objects and characters to lighting design. The design of the game space can be understood in terms of *mise-en-scène*; in case of game text, *mise-en-scène* blends the interactive and the narrative aspects of the game space (Pigulak 2022: 110). This role of *mise-en-scène* overlaps to a certain degree with the function of the narrative design, wherein it focuses the player’s attention on a specific object, part of the set design, or character; just like narrative design, *mise-en-scène* can be used to organize the space of the game, giving prominence to objects crucial to the narrative and interactivity of the game (Pigulak 2022: 110-111).

Interestingly, *The Medium* makes heavy use of this function of *mise-en-scène*, but not in all of its locations. At the beginning of the game, just before Marianne receives her literal “call to adventure”, in the form of a phone call summoning her to the abandoned Niwa resort, the player has the opportunity to explore the place where she lives. The tenement apartment, which belongs to her deceased legal guardian, is full of his belongings and memorabilia related to his past ^1^. The player, controlling Marianne, can pick up and examine numerous items around the apartment; Marianne’s comments regarding the objects she looks at reveal information about Jack’s past and the relationship she had with him. This sequence is probably the most detailed and oriented towards encouraging the player to explore the environment. However, once Marianne arrives at Niwa, there are few to no objects essential to the storyline concerning her family history.

Another important feature of game spaces are evocative spaces and elements, which tend to draw upon the player’s metagame knowledge and meta-genre knowledge. Relying on the already existing narrative-related experiences, the players infuse the evocative spaces and other elements with significance through reading and connecting them (Nitsche 2008: 44). Evocative spaces are also closely connected to environmental storytelling, which depends on using the virtual environment as a tool to convey the story, accentuating smaller stories expressed by the very world of the game ( Markocki 2021: 72-73).

*The Medium* features detailed environmental storytelling concerning the Niwa massacre, but very few evocative spaces related to Marianne’s family history. There is not much in the game space for the player to infuse with significance, and practically no instances of pull narrative. Initial conversations with Sadness give the player no clues regarding her connection to Marianne. In a story focused on family trauma the player would expect appropriate evocative elements to be incorporated into the game world to stimulate the meaning-making process (Nitsche 2008: 44). However, *The Medium* lacks these crucial elements, which makes the comprehension of family history (i.e. the most critical part of *The Medium’s* story) difficult; instead, the aftermath of the Niwa massacre constitutes the foundation of the games environmental storytelling and artistic choices.

The sequences in the Spirit World demonstrate how it literally allows the past and present to coexist in the game—both in terms of gameplay and story—seeing as Marianne can interact with remnants that continue to exist but belong to the past inside her present timeline. It is also important to note that despite the visual (re)presentation, the material world and the Spirit World are not, in fact, two separate worlds; similarly to *Silent Hill*’s Otherworld, the Spirit World is rather a distinct version of the same depicted world occupying the same space within the same game world, which can be readily distinguished by the vastly different aesthetic design, interactable parts, and now traversable areas ( Bizzocchi and Tannenbaum 2011) within the confines of the same virtual environment. Appropriately, the time and space in *The Medium* do not merge, but overlap, the past being always superimposed on the present. The primary location explored by the player, the abandoned Niwa resort, is both the most artistically polished game space, and the most saturated with details as far as the Spirit World is concerned. While the virtual environment of the Niwa resort in the material world offers the richest storytelling, including not only indices pointing to the murdered guests, but also to the previous everyday functioning of the resort as well as to the investigation carried out in it after the massacre, the Spirit World in that location introduces actual spirit entities and remnants of the past. The player can examine what remains of the Niwa resort, but they can actually interact with spirit entities and remnants to further explore the past.

As the game progresses, the story and the gameplay shift away from the Niwa massacre to Marianne’s family history. Consequently, the emphasis of the early gameplay is placed on clear objectives and actions required to achieve them—searching for the man who made the phone call to Marianne, helping the spirits of the massacre victims to move on, and unraveling the mystery of what had really happened at Niwa. However, as the narrative progresses, the Spirit World shifts from being a place of exploration to being a space of encounters and introspection. Marianne discovers that the man who called her, Thomas Rekowicz, and his daughter were the focal point of the events that led to the Niwa Massacre; she also discovers that they were her father and elder sister, respectively. By interacting with and talking to the Childeater or the Hound, who remained trapped on Niwa’s grounds, Marriane gathers evermore information, allowing the player to sort out the past events. The second part of the game is therefore more concerned with introspection and understanding Marianne’s origins and fate. This means that the nature of the challenges the player was expecting when they started the game, and responding to in the first part of the game, changes, altering from skill and dexterity based ones to cognitive and emotional ones ( Cole *et al.* 2015). This can lead the player to shift their attention and engagement away from visual and ludic aspects of the game towards the story and the people it features, resulting in greater character attachment ( Bopp *et al.* 2019), and to focus on eudaimonic gratification. However, in practice, the past ultimately eclipses the present, and the main character of the game is gradually overshadowed by other characters whose complex personalities and traumatic backstories demand increasingly more attention, judgment, and emotional responses from the player. As Marianne—together with the player—is relentlessly bombarded with more horrifying details and appalling secrets of the people who were strangers to her before she stepped into the resort but are now revealed to be somehow connected to her either through blood and love or through assault and hatred, the player learns nothing new about Marianne herself. At this point in the game, the story—and the narrative—is concerned with the past alone. Marianne has very little internal monologue that would shed light on how she is dealing with all the new information. If a comparison were to be made to a character in a similar situation, Heather Mason in *Silent Hill 3* was also faced with discovering that there was much more to what she thought was her life, and had to quickly gain extensive intimate knowledge concerning the cruel, twisted history of family and her own birth before she had been, much like Marianne, taken away from a dangerous place by someone who wanted to protect her. There is much that Heather must understand and accept: the fact that she was previously another person, a young girl named Alessa, and that this girl was in so much pain due to the actions of her fanatical mother that her only wish was to die and thus escape her suffering. After the confrontation with the Memory of Alessa, Heather mentions how it is strange for her to refer to her former self as “Alessa”, since they are the same person, and adds: “‘You’ and I don’t think alike, after all … And it’s not that I don’t remember that sick room either …” ( *Silent Hill 3*, 2003). In this way, Heather confirms what the player could previously only infer from her actions and spoken lines: the way she feels, what she thinks about her circumstances, and what her intentions will be as she continues her journey. In *The Medium*, Marianne does nothing of the sort, forcing the player to either guess or ignore her feelings and motivations. Such design strips her of subjectivity, rendering her experiences and feelings inconsequential. In practice, this means that for the player, who is in control of at least some of her actions, there is very little difference between Marianne as a character at the beginning of the game and halfway through the game.

This situation is, once again, consistent with the story told by *The Medium*; while the narrative of the game revolves around Marianne, the tragic story the creators attempted to convey concerns Thomas Rekowicz and his anguish over his powerlessness to protect his daughters. Marianne is more of a spectator than an actor in this drama. The push narrative ^2^ continually provides the player with new information in order to sustain immersion in lieu of allowing them more agency; in these circumstances, Marianne continues to be the avatar, but never becomes the main character. Her role is limited to that of a high quality exploration tool. And even if this choice is fitting in regards to the narrative design of the game, it takes away from the game mechanics available to the player.

*The Medium* features an uneasy balance between its ludic and narrative elements, which continues to shift in favor of the narrative as the game progresses. The more justified the reasons for Marianne’s exploration of the depicted world, the fewer the tools that the game offers the player to influence the situations they participate in and their outcomes. This strategy does not seem to be directly aimed at player’s agency, or even constitute an attempt of subverting that agency, but rather is a logical outcome of adopting a particular hierarchy of narrative tools atypical to the genre, like hard scripting and foreshadowing (in cases where horror games would frequently opt for emergent story and soft scripting as narrative techniques). This seems to be confirmed by—among other things—the way the non-playable characters ^3^ are structured, which conforms to the rules of quest design, but is used in a way more specific to gamebooks. ^4^

As a work of art *The Medium* is direct and explicit in its way of evoking an emotional response in the player. Despite the game’s tactful handling of its most sensitive moments, like the scene in which Thomas Rekowicz flips through Richard’s sketchbook which documents his abuse of Lilianne, or the way the game explains Henry’s childhood and twisted logic, it could still be argued that the characters themselves are presented very narrowly through the optics of their one specific role ( Howard 2008: 71-72). In gameplay, on the other hand, *The Medium*’s directness manifests in its conspicuous linearity. The explicit nature of the storytelling entails the game’s reliance on push narrative and extremely tight narrative design, where the player’s movement and the amount of information they obtain is controlled very strictly. The use of horror convention, in which the game fantasy is realized through “cause-and-effect relationships suggested by the sequence of facts [that fantasy] details” ( Govil-Pai 2006: 68), fulfills the function of a classic quest system, which results in the constant overlapping of the material world and the Spirit World. Consequently, the gameplay involves multiple timeline shifts to the point of obliterating the boundary between past and present, as mentioned previously. Even if the mid-game is structured like a typical Trial Narrative ( van der Meer 2019), the use of classic cause-and-effect sequence (cf. Stepnowska 2017: 17-18) to represent the fantasy in the game means that between the linear delivery of new information and the rigid game progression the main questline of *The Medium* gradually becomes more and more like a railroad (cf. van der Meer 2019).

As a result, the sequences depicting the events of the past in which the player is in control of Spirit Thomas create an attractive break from this rigid progression. Due to his internal monologue and distinctive, cynical personality, Spirit Thomas may come across to many players as a more fleshed out, believable character, if not just more interesting in regards to his gameplay sections—even if he is available as a playable character only in a few selected sequences. The gameplay itself might also seem more satisfying whenever the player plays as Spirit Thomas, partially due to the novelty of locations within Richard’s mind and Henry’s mind—complete with new aesthetics, dangers and enemies—and partially because of the way in which the progress is rewarded with new information. It is also important to note that when the player takes control of Spirit Thomas, they already know the results and consequences of that character’s actions, even if those parts of the game create fully-fledged artistic experiences. It could be inferred that the sequences which employ Spirit Thomas as an avatar can be very satisfying immersive experiences for the specific type of players known as Immersion Seekers—including both subgroups—in accordance with the Gamer Motivation Model ^5^ ( Our Gamer Motivation Model n.d.). In the context of the game’s plot, specifically the artistic arrangement of the order of exposition, the character of Spirit Thomas appears to be more coherent and credible; for the Fantasy players—again, in accordance with the Gamer Motivation Model—in turn, his role as avatar offers a new perspective on the gameplay and a welcome departure from the previous gameplay model.

### The performance and experience

There are many factors that both influence and, occasionally, constrain the player’s performance as they play the game. The traits of Marianne as a player character that are considerably distinct from the standard avatar characterization indicate the manner in which *The Medium* approaches the matter of the user experience design. From the designers’ perspective, the player gets immersed in the story rather than their own decisions, and Marianne’s essential function as the narrator of the game is continually reaffirmed by many elements included by game development (such as her hesitance to take a stance regarding other characters’ behavior, or the inner monologues that serve only to emphasize the importance of particular information rather than her emotional growth). Additionally, the development solutions and animations of the main character clearly indicate which mechanics and visuals Bloober Team deemed to be crucial to conveying the story. For instance, Marianne may run into objects or even occasionally fail to bend her knees, but her animation of walking down the stairs is outstanding—which for a mid-budget production developed with Unreal Engine 4 is quite a development challenge—especially in moments crucial for building tension or ones constituting the metaphor of a rabbit hole. Both the cut-scene animations and the technical level design are, in this context, created with the utmost attention to detail, matching the standards of a work of art. In effect, *The Medium* delivers an impressive performance on its own while leaving little room for the players to perform. Even when the player has the chance to interact with the game world, they choose an item from a pop-up list when deciding on the possible interaction with a specific object (e.g. opening a lock). This solution might have been a standard one for story-centered games in the early 00s, but now prevails only in simple games (like hidden object or arcade games). This would place *The Medium* in the “wandering-through-story” category, as Jon Bannister calls it ( Spigel 2005: 84), rather than one where the player shapes the story themselves. Of course, the above remarks by no means constitute a criticism of development solutions but are merely observations concerning the clearly well-defined priorities of the creative team.

As it is, the game tempo of *The Medium* is contingent on suspense and the player’s, and not the gameplay, progression. The puzzle quests themselves allow the player to tame the depicted world, but the lack of any progression of their difficulty level creates the impression that the given questline expresses itself like a walkthrough, providing the player with information essential to the actual gameplay. This further contributes to the impression that the focus of the game revolves around the game art, with history playing an important role, while playability and gameplay—not so much. In terms of game design, as a result of its treatment of agency-expressing mechanics and their influence on the pacing of the story, *The Medium* appears to be an illustration of how not to pace a game.

The nature of this problem is also manifested in the fact that *The Medium* does not feature any actual boss battles, while there are sequences in the game which can be said to go against the player’s expectations in regards to the horror genre. An early example of this is the confrontation with the Childeater. This scene occurs after Marianne has learned what Richard did to Lilianne. Marianne and the Childeater trade some bitter words, accusations and excuses, all the while Marianne is held down by the Childeater’s slimy limbs. The audiovisual expressive vocabulary of the scene includes wide shots of Marianne restrained in the wheelchair, with more of the enemy’s tentacles slithering ominously behind her, juxtaposed with mid-shots and close-ups of Marianne’s face contorted in anger, hostility and disgust; the images are accompanied by unpleasant diegetic sounds and suspenseful music. This entire sequence, however, culminates in the following exchange:

MARIANNE: This man … Thomas … Who … What is he?

CHILDEATER: He is the one who butchers the soul. The one who breaks it. But you … Yes, I know you … You can set me free … I can feel it. Please, do it.

MARIANNE: You don’t deserve it. You deserve nothing! Nothing, do you hear me?!

CHILDEATER: Then give it to me. Please … To not exist. That’s all I want.

MARIANNE: So be it. ( *The Medium*, 2022)

With those words, Marianne opens her left fist, releasing a bright light which envelops the Childeater, herself, and eventually everything else, leaving the player looking at a blank screen, possibly pondering the Childeater’s final farewell: “Thank you, the girl from the red house” ( *The Medium*, 2022). There is no confrontation, no satisfactory resolution from the gameplay point of view, despite the fact that the entire sequence is built up in a manner which the players can easily interpret as a prelude to a boss battle, based on their metagame knowledge.

The same issue comes into play later in the game, as *The Medium* offers no possible closure of the story- and gameplay-centered conflict introduced by the character of the Maw. From the ludic perspective, Marianne starts out with an objective and a series of quests: she is supposed to explore Niwa and grant the dead rest by sending their trapped spirits away. During this part of the game, the player becomes aware of a dangerous enemy—the Maw, a powerful hostile spirit entity that not only roams the Spirit World, where Marianne needs to regularly evade it by means of a couple of stealth mechanics, but also is able to partially exist in the material world. The ludic and aesthetic presentation and behavior of the Maw suggest to the player that they might at some point expect either a confrontation with that character—a final boss battle—or at least a final chase sequence, in which they could slip the Maw’s grasp once and for all. However, as Marianne’s work, represented in the gameplay as puzzles, is replaced with the preparation for what the player may presume to be her ultimate task—saving Lilianne—the player, by now emotionally invested, is denied more and more agency within gameplay, as well as, ultimately, a satisfying resolution of the story. Confronting the Maw—either by attacking it with the provided weapon or by stealing Lilianne away from its reach—is not an option available to the player. The lack of availability of interactivity thus translates into a lack of possibility of facing the main antagonist and only a number of lackluster interactions, which are few and far between. The Maw is supposed to be the primary antagonist, the evil haunting the site of the massacre, and the greatest threat to Marianne herself, seeing as it single-mindedly hunts her to take possession of her body. And yet, in the game whose scope ( Bizzocchi and Tannenbaum 2011) can extend from ten to twelve hours, according to the official website, the total time of all interactions between Marianne and the Maw, taking into consideration every instance in which the two occupy a single location, spans only around eighteen to twenty-four minutes, depending on the player’s strategy and reflexes. Even taking into consideration the symbolic significance of the Maw, the gameplay does little to heighten, defuse, or resolve the tension between Marianne and the Maw in a meaningful manner that would be engaging to the player and fit the overall pacing of the story.

The aforementioned abrupt change of objective of the gameplay and the emphasis of the narrative creates another problem worth commenting on in this article. To better approach this matter it would be useful to consider two concepts related to aesthetic of reception: Eco’s Model Reader ( Eco 1984) and Jauss’ horizon of expectations ( Jauss 2005). Both of them can aid in examining the game tempo of *The Medium*—more specifically, the tension between the pacing imposed by the game and the pacing preferred by the player. Assuming that “every text is made of two components: the information provided by the author and that added by the Model Reader” ( Eco 1984: 206), and taking into consideration that “the text postulates the presumptuous reader as one of its constitutive elements” ( Eco 1984: 206), the gameplay is founded on the constant tension between the way the game progresses, telling its story through the narrative design the Model Player (Reader) would ideally follow and explore, and the preferences and expectations of the actual player, which contribute to that player’s horizon of expectations. This is a position unavoidable as far as the process of consuming any text of culture is concerned, seeing as

the new text evokes for the reader … the horizon of expectations and rules familiar from earlier texts, which are then varied, corrected, altered, or even just reproduced. Variation and correction determine the scope, whereas alteration and reproduction determine the borders of a genre-structure ( Jauss 2005: 24).

The horizon of expectations emerges from what Jauss describes as the horizon of experience of the audience—in the case of a game text, the player; the horizon of experience consists of the entirety of the player’s previous experience gained from consuming other texts of culture, which builds their knowledge of conventions, structure, composition etc. that is then applied in the current process of gameplay. In case of games, this knowledge is particularly important due to the fact that a game “demands specific actions from the player for progression to occur”; this “contractual condition between game and player,” in Krzywinska’s words, differentiates the process of consuming a game text from the process of consuming texts of other media and, more importantly, profoundly affects the way in which the player expects the game to realize its potential and exert its effect ( Krzywinska 2009: 270). In the case of a horror game, the player will have certain expectations regarding the manner in which the horror is going to be delivered, even if some of those expectations might be influenced by the expressive characteristics and narrative format of cinema, both of which tend to permeate the majority of the texts of the horror genre ( Krzywinska 2009: 270). The same contractual condition, situated well within the player’s horizon of experience, will affect their playthrough of *The Medium*—while the game’s tense atmosphere, wide shots and particular camera angles, together with the overall aesthetics, will be readily accepted, if not welcome by the target audience, precisely due to their previous experience with other game texts of that genre, the same experience will surely render the lack of closure and confrontation as jarring and inadequate. ^6^

The horizon of experience is also a component of what Bourdieu refers to as cultural capital—in other words, the “familiarity with the internal logic of works that aesthetic enjoyment presupposes” ( Bourdieu 1996: 2). In the particular case of games, the cultural capital of the player—which marks their horizon of expectations—is not limited to their familiarity with the genre, or other games belonging to it, but encompasses the knowledge of the world beyond the text itself: the history, geography, religion and politics, as well as the art of the country which is the point of reference for the game world. It is that knowledge which has the potential to shape the distinctive perception and interpretation of the signs, indices and symbols disseminated throughout game world, all of which in the case of *The Medium* refer to discernible aspects of Polish reality such as customs, the experience of the socialist era in Polish history, and religiousness. The same knowledge can invite the player to pursue passing references, indirect mentions, or intertextual connections included in the game, regardless of their actual function in the gameplay. Following and collecting cultural symbols which permeate the world of *The Medium* affects the pacing of the gameplay, which is then oriented more towards the search and scrutiny of cultural indices and signs that allow the player to embed the narrative and gameplay in the extratextual context than towards following the narrative design from one event to another. It is also likely that for the global player community the better part of the multitude of the signs and symbols mentioned above, largely inaccessible to people who lack the intimate knowledge of the genre, as well as of the Polish pop culture of the 80s and 90s, may be difficult to decipher and therefore bear little significance for their experience and interpretation of the game.

Another issue worth discussing and one that is quite peculiar from the designers’ perspective is the manner in which *The Medium* uses the inertia of player choices. The emotional gratification is directly bound to the unfolding story rather than to consequences of players’ decisions in terms of gameplay, as described earlier. For the purpose of this article “inertia” will refer to the distance between the player making a decision on whether or not to take action, and the game world feedback concerning that particular decision, measured in the number of decision nodes between the decision itself and the feedback. Inertia is one of the factors influencing the gameplay tempo, and can signal the extent to which the game world is open and developed. *The Medium* features only a few nonlinear moments of exerting the player’s agency throughout the game, and the ones available affect only the immediate gameplay experience. And although it is inconsequential to discriminate between “superior” and “inferior” inertia, *The Medium* gameplay is paced by means of tools associated with cinematic texts rather than game texts, which means that the nature of inertia encountered in the game resembles much more the kind one would expect and appreciate in a film text. ^7^ The game steers the player through its world through a cinematic narrative format and expressive apparatus, rather than engaging narrative design. This leads to an effect opposite of the one that would be normally desirable in the case of a game text—not only does it reinforce the impression that the puzzle quests are of no consequence in the context of the game as a narrative (the necessary hints are always nearby and do not require an extensive knowledge of the history of the Niwa resort or the personalities of the characters crucial to the story), but also it the changes the game tempo in favor of reactive elements at the cost of interactive ones. More specifically, the emphasis shifts from the operationalizable decisions of the player to the aesthetics, specifically the audiovisual representation. This shift increases the ludonarrative dissonance since the rate at which the story unfolds speeds up regardless of the player’s participation; at this point, the player is not so much making choices that would be rewarded with a noticeable change of the gameplay pace, as being pushed along by the plot.

This situation additionally affects the player’s perception of choices themselves, which by now appear binary as informed by the previously mentioned trial narrative structure. *The Medium* includes only one ending proper, without any flavors that would influence the experience of that ending (in contrast to, for example, the possible ending flavors of *Silent Hill 2*: “Leave”, “In Water,” and “Maria”). In contrast, there are at least five game-over scenarios, all of which put an end to the gameplay before the final resolution. Some of them include being captured by the Maw in the Spirit World or in the material world, or being overwhelmed by spirit moles; in both cases the player loses their avatar as the protagonist dies, thus failing the game. The game-over scenarios for the protagonist in *The Medium* are fairly typical for the horror game genre. Meanwhile, the other game-over scenarios concern a different avatar—Spirit Thomas. In the sequences where Marianne’s visions show her the past, the player relives fragments of Thomas Rekowicz’s life, which means they have the opportunity to control Spirit Thomas. The player can fail the game by getting Spirit Thomas killed in Henry’s or Richard’s mind, if they cannot avoid the dangers of those virtual environments. These moments, despite being only game-over scenarios, seem more relevant to the plot of the game, and can reinforce the positive negative experience. Unfortunately, none of the game-over scenarios constitute sufficient feedback in terms of gameplay since they do not equip players with information concerning the most crucial choices resulting in failure; as such, the players’ self-actualisation is limited, and while such solution could be understandable in a game aiming for high replayability, in *The Medium* it is not justified by any learning value. As a result the player knows they are limited to one, “correct” path, even if this was not the deliberate intention of the game development team.

The above issue is probably related to the emphasis on the artistic value of the final product. The visual aspect of level design in *The Medium* is artistic to a high degree, which corresponds with the game’s clear aspirations; the recreation of Hotel Orbis “Cracovia,” the clear inspirations drawn from Beksiński, as well as the impressive performance by Rosati and Dorociński, all confirm that *The Medium* appears to be primarily an art-driven experience, featuring a story that the player follows, instead of leading it (cf. Jenkins 2004). The game aesthetics (in a broad sense of the term cf. Schell (2008)) come together to create an impressive work of art at the cost of the game’s interactivity. ^8^

Linear stories in speculative game texts are not uncommon. In well-balanced games the linear nature of the game’s main story is rarely an issue—the majority of the games mentioned in this article tend to rely on narratives that unfold in a more or less linear manner. Even if the game offers the player only one specific way to progress through it, and only one specific ending, the narrative experienced by the player is not a singular entity (Nitsche 2008: 45). The player needs to comprehend the game world and whatever occurrences or encounters which take (or have taken) place within it—the context and significance are determined by the player, contributing to the meaningfulness of the resulting experience. As Nitsche also points out, “[w]hile the reader of a novel is limited to the given text, the player of a game interacts with [the] evocative elements, cocreates them, and changes them. Whatever manifests itself in the shape of this comprehension is of a unique nature.” (Nitsche 2008: 44-45). In *The Medium*, meanwhile, one can readily observe the underlying drive to generate strong emotions through narrative elements rather than through interaction or particular game mechanics. The story of the game is meant to elicit complex negative emotions in order to exert its effect, a crucial part of which is the player’s appreciation of the sad narrative, involving “tender feelings and, more precisely, feelings of being moved” ( Cova *et al.* 2017: 356). As such, the dominant narrative of *The Medium*, with its themes of loss, sacrifice, abuse, and anguish, aims at producing a specific “blend of cognitive activity … and affective states” ( Cova *et al.* 2017: 356). As the player progresses through the game, experiencing and performing the unfolding story, they are meant to “reflect on meaningful questions” and experience “the feelings of being moved” ( Cova *et al.* 2017: 356), thus achieving a sense of eudaimonic gratification associated with art—perhaps this is why the narrative in *The Medium* appears as decidedly linear. As a result of this ambitious premise, the game attempts to make meaning by engaging the player with emotionally powerful narrative moments, expressed in the form of aesthetically and visually captivating cut-scenes. The camera movements and angles used during those cut-scenes are meant to establish a poignant, distressing or sorrowful atmosphere, and, together with music, signal the significance of the events on screen in the context of the entire story of *The Medium.* A memorable example of this is one of the game’s most moving scenes, in which at the end of the game Marianne finds Spirit Thomas, the only remnant of her father, confined in the special bunker; the very first words of Spirit Thomas are those of concern:

SPIRIT THOMAS: It’s you. You’re alive.

MARIANNE: Thomas?

SPIRIT THOMAS: in a way. Yes.

MARIANNE: Wait, you’re the other one. The spirit. But the Hound … I thought you were gone.

SPIRIT THOMAS: Gone? No. Trapped. ( *The Medium*, 2022)

This scene actually constitutes the beginning of the game’s story as told by Marianne up to that point, both through cut-scenes and her narration throughout the game. The player can immediately realize that they are about to not only witness a conclusion to an important story element of the game, but also be subject to the full emotional impact of this particular narrative component of the game, which the story so far encouraged them to engage with. The meeting of Marianne and Spirit Thomas is a scene that is both satisfying to witness and sorrowful to experience, taking into consideration that the protagonist just missed the opportunity to meet Thomas Rekowicz by so little in the context of space and time in the game world. As such, Spirit Thomas, despite being evidently different from Thomas Rekowicz, takes on the role of a father figure for Marianne, who is the baby that had been left behind, and for the player, who is not only the actor, but also the spectator. Wide camera shots are replaced with mid-shots, and then close-ups, as the music swells at the very end, when Spirit Thomas assures Marianne that Thomas Rekowicz loved both his daughters very much. When eventually Spirit Thomas urges Marianne to flee, so that he can delay the Maw and guarantee her safety for at least a little while, Marianne is too emotionally attached to simply leave him behind:

SPIRIT THOMAS: It’s coming. Time for you to go. I’ll hold it off for as long as I can.

MARIANNE: I’m not leaving you!

SPIRIT THOMAS: You can’t always save everyone, butterfly. Trust me, I know. And … I’m sorry. ( *The Medium*, 2022)

Immediately after this apology, Spirit Thomas pushes Marianne out of the Spirit World against her will, and away from himself so that he can stand between her and the Maw, presumably sacrificing himself for her. Thus, in such a relatively short but undeniably touching sequence—where last horizontally split shot shows a close-up of Marianne’s face in the material world, reflecting complex feelings as Spirit Thomas holds her hands and calls her “butterfly”, and a close-up of Spirit Thomas’s soft smile as he looks at her— *The Medium* elicits a whole range of complicated, predominantly negative affective reactions for the player to contemplate and attempt to reflect upon. This moment is evidently one of the few in which the creators seem to have shifted the emphasis from interactivity and agency to expression of powerful concepts and emotionally engaging experiences.

### Agency and fixed experience

As the art overtakes the story’s place, one can deliberate on the moments in gameplay that benefit from this approach and the ones that lose some of its quality because of it. It is worth emphasizing that—very uncommonly for the genre— *The Medium* foregoes the importance of the narrative in favor of its art as an informed decision. The game director, Wojciech Piejko, stated openly that the team set out to design “the game like a movie, planning the best shots, the best camera angles” ( Wales 2020). The decision to prioritize the cinematic aspects of the game results in decreasing *The Medium*’s potential as a meaningful interactive experience, sometimes even making it present itself more like an interactive fiction movie than a game text.

In games, there exists a necessity for “gateways between player and game system”, in the form of game interfaces (Nitsche 2008: 33). Players can interact with game spaces in two ways; one of them is movement through a space. Movement dominates the gameplay of *The Medium*, as Marianne traverses the space of Niwa in the material world and in the Spirit World, collecting clues and items. She then sometimes uses those items and navigates the two worlds separately, thus engaging in what Nitche refers to as “specialized manipulation of elements within [the] space” (Nitsche 2008: 33). In this way the player interacts with the game system and is rewarded with more pieces of the backstory, but such solution grants little satisfaction in terms of influencing the emerging narrative, or understanding Marianne’s motivations, since the backstory improves the player’s comprehension of the past–and even some of the present–events, but does not establish Marianne as a believable central character exploring those events.

This peculiar backstory of the game world, combined with the prevailing push narrative and limited ambiguity, leads to a very strained ending. Since everything is either shown, disclosed, or explained to Marianne directly, sometimes more than once (e.g. the origin of the Maw is suggested through echoes of the past in the Red House once, and then explained again twice by Spirit Thomas and later Lilianne herself), the game places little emphasis on the player’s interpretation of the information and active inference. There are simply not enough evocative narrative elements in the form of either situations, scenes or items that would “support and possibly guide the player’s comprehension” of the narrative; as Nitsche explains,

[the] elements’ task is to improve a player’s experience and understanding of the game world. Players encounter and read these elements, comprehend the information in the context of a fictional world, and learn from them as they build contextual connections between elements (Nitsche 2008: 37).

The narrative of *The Medium* does not require the player to build any contextual connections. The story is instead pushed unto the player, and it seems to start and end without needing any player’s input. As a result, the gameplay merely supports the story, as it explores itself in front of the players, not because of their choices or actions. As Marianne arrives at the pier at the lake from her dreams, looking for her sister, the player has yet to make any significant connection, discovery, or choice of their own. Upon finding Lilianne, the sisters have a lengthy conversation, during which Lilianne fills in any of the gaps in Marianne’s—and the player’s—understanding of what had taken place in Niwa. During this cut-scene Lilianne, clearly depicted as exhausted and filled with anguish, gives Marianne a gun, asking the protagonist to free her from her agonizing existence by ending her life, since it is impossible for Marianne to send the Maw away as long as Lilianne lives:

LILIANNE: It all ends in me.

MARIANNE: But … No … NO!

LILIANNE: You can’t send a spirit away while the host is still alive. That’s why you couldn’t destroy the monster. That’s why Sadness didn’t want to go.

MARIANNE: But … You’re my sister!

LILIANNE: That’s why it has to be you. I— I’m not strong enough. It won’t let me. Only you can end this. Only you can fix what our father could not.

MARIANNE: Lilianne … I can’t. Please don’t make me do this.

LILIANNE: I’m sorry. It’s the only way to destroy it. To prevent further bloodshed. ( *The Medium*, 2022)

At this point, the Maw reaches the two sisters, proclaiming Marianne to be its perfect host; it continues to threaten her, dismissively referring to Lilianne as “old skin suit”, while Lilianne herself continues pleading with Marianne to “set her free”. The player can see Marianne putting the gun to her own head and threatening to kill herself, aware that the Maw needs her body to leave Niwa. The scene, from the moment of the Maw’s arrival, is shown in vertical split screen. This time, however, the single point of view depicts single subject; depending on the characters’ movements and the camera movements and angle, the players can see Marianne and other characters as they are in either the Spirit World, which this time occupies the dominant (left) part of the screen, or in the material world. The cut-scene constitutes the actual end of the game, never allowing the players to make the choice they would naturally expect at this point of gameplay, based on their metagame knowledge. Instead, in extreme close-up, Marianne opens her eyes, the screen goes blank, and the player can hear a single gunshot.

In this way, the game deprives Marianne—the main character of the story and the player’s character—not only of her present, but also of her future, regardless of her role as a player character (no choice is offered to the player) or as a character proper; she quite possibly ends her life in this moment, and if she lives, the game is not interested in her fate enough to inform the player of it. This radical artistic and design choice would fit a different audiovisual medium text, such as a film or a TV series, but falls flat in the case of a game—incidentally, this is an impression shared by the players, as numerous users (over 40 Steam reviewers, among others) believe that Marianne resembles a movie character rather than a typical game protagonist. *The Medium* undoubtedly excels at creating what Jørgensen refers to as “positive discomfort” ( Jørgensen 2019: 155). The game follows a script resembling very much that of a film matching the conceptual and aesthetic horror convention, enriched with attributes of interactivity. In a broader perspective of the pleasures of horror, it is worth considering the distancing factor in various artworks—be it literature, film, or game texts; according to Crowther, distance is what “momentarily invests the object with the character of representation rather than that of real physical existence” ( Crowther 1993: 123). With this in mind, it is conceivable to reason that there is a purpose to the way Marianne is objectified by the game’s storytelling; the fact that her character is instrumental, inert in terms of agency (both her own and the one she would provide to the player as an avatar), and stripped of subjectivity facilitates the creation of an atmosphere of horror and tension without increasing the stakes concerning the game progress. In fact, a common complaint among the players (in this case, the Steam reviewers among others) is the lack of tension build-up, as well as Marianne’s failure to make them feel that they are making progress in the game. Playing *The Medium*, the player can experience the “shudders and shocks”, as Crowther notes—or, in Burke’s words, “delightful horror, a sort of tranquillity tinged with terror” ( Burke 1999: 123)—of a horror scenario “without running for the exit, because [they] know that the frightening phenomena are representations rather than realities” ( Crowther 1993: 123). And yet digital games are unquestionably a medium that is known to permit “the sense of safety to be challenged both on the level of fiction and on the level of play”; this translates into their capacity for creating positive discomfort ( Jørgensen 2016) which reaches beyond the representation and mirroring fright and unease, allowing for dissonance, impotence, and opposition as well. The players can experience discomfort in a game—either positive or negative—either as a sense of frustration stemming from being prevented from taking action they would deem as “right” or necessary, and instead “being the victim of the bad decisions made by nonplayer characters” ( Jørgensen 2019: 159), or as a result of the sense of complicity ( Isbister 2016: 8-10) caused by the “feeling that the events that unfold in the game happen because of the player’s choices” ( Jørgensen 2019: 158). Regardless of whether it is the player’s agency or the very lack thereof that leads to the discomfort, the deliberate attempt to cause displeasure in the player is meant to inspire their reflection ( Jørgensen 2016), contributing to the eudaimonic gratification they might seek. Positive discomfort is often employed in speculative games, especially in the horror genre, but the manner in which it is implemented in *The Medium* is far from optimal ^9^. While it is a common technique in horror games to evoke a feeling of discomfort by limiting the players’ agency in certain moments, *The Medium* implements it in a peculiar way; the oneiric Spirit World would appear to be the natural environment in which players’ feeling of control over the avatar (and the gameplay itself) could be limited, but it is actually the material world in which the player repeatedly feels restricted by the set of actions possible for Marianne. This perspective is frequently represented in Steam reviews.

In their research on the legitimacy of causing discomfort to the player, Gowler and Iacovides created a research tool taking into account various aspects of game design that participate in causing discomfort ( Gowler and Iacovides 2019). In a similar manner, for the purpose of this article, 5,292 Steam user reviews have been examined ^10^, all written by people who purchased the game. Among 719 unambiguously negative reviews, 80 mentioned the lack of agency, and 177 alluded to the ludonarrative dissonance. The users commented on the gameplay sophistication lagging behind the artistic aspect of the game, as they rated the game’s elements as graphically appealing, with a decent story and nearly non-existent gameplay, or claimed that it was easy to realize that the repetition of some elements is just a placeholder to create the illusion of a bigger gameplay.

Of course, random individual comments do not reflect the reception of the game by the mass audience; however, a Steam review deemed by the community as one of the “most helpful” ones does summarize the majority of the problems of *The Medium*, describing it as a disappointing walking simulator-like experience, dressed up as a horror game, strongly emphasizing that it lacks game-like experience.

The leisurely paced endgame gameplay, intended to give the player time to appreciate the aesthetics of the game, might have been indeed, in the optics of game design, “enriched” with puzzle quests during the late stage of the game development. The players’ reception of the puzzles featured in *The Medium* appears to reflect a similar idea—the majority of reviews (1022) is rather neutral, pointing out their low difficulty level. Nonetheless, many reviews (677) emphasize the illusory nature of the choices involved in the puzzle solving, or even—according to some (410)—their pointlessness. Another Steam review labeled as “most helpful” highlights the frustration caused by the puzzle design, triggering the feeling of being treated by the game in a very condescending way by offering a single story with a single ending, forcing the player to push ahead in one direction only and, whenever the player stops in a location to solve a puzzle, by taking the players’ agency away, without offering the possibility to fail, assess one’s mistakes or discover the solution on their own.

On the other hand, the main struggle, namely gameplay sequences involving the Maw, is reduced to an obstacle quest with wasted potential—both in terms of the resolution of the narrative conflict and engaging gameplay. The game creators might have quite possibly aspired to introduce what Hopeametsä calls “positive negative experience” ( Hopeametsä 2008: 191). This type of experience would be a good fit for a live game where the players are able to aim at a personalized in-game resolution; however, in a single player game, it clashes with conventional understanding of meaningful play Salen Tekinbaş and Zimmerman (2003). In fact, the common reading of the term “positive negative experience” as proposed by Montola (and referred to by Hopeametsä) implies the possibility of frustration—even one caused by game rules—being the catalyst of a personalized narrative experience.

Whether the introduction of the positive negative experience does indeed meet the expectations of the target audience in the case of *The Medium* is a question worth considering. According to Montola, the players

belong to a subculture of gamers that is convinced of the value of non-fun games. They aim for intense experiences, regardless of their supposed emotional valence, and for them, the value of negative emotions is larger than just giving meaning to the subsequent positive twists ( Montola 2010: 7).

The number of Steam reviews, both utterly positive and utterly negative might suggest that the positive negative experience employed in *The Medium* does resonate with some of the players, but antagonizes others. Additionally, the manner in which the Bloober Teams implemented the positive negative experience appears to support this assumption. The focus on the almost theatrical take on the artistic aspect of the game at the expense of the experience of agency and gameplay, even in spite of the possibility of frustrating the players, might not necessarily be a mistake of any kind, but simply compliance with the positive negative experience trend specific to aesthetic-centered games (cf. Umbelino and da Mota 2021).

## Conclusion

As we include game texts among other works of art we need to take a closer look at their specific traits and affordances. There are game texts that aspire to touch upon serious sensitive subject matter; in their ambitious attempt to do this in a way specific to the game medium, they build upon works of art of other media, but make use of unique tools available only to their own medium. In the case of digital games the point of contact between the artist intention and the audience experience is just as important as in the case of any other work of art. This article has used as its case study *The Medium*, a game doubtlessly polished and well thought out as far as its artistic value is concerned. The creators of *The Medium* made it clear in interviews and other materials that they wanted their game to convey a certain important message. However, as Rothschild and colleagues point out, “the moment a game is available for an audience to play … control passes out of the hands of the designers of the experience and into the hands of those who take that experience and bring to it their own desires, ideas, and interpretations” ( Rothschild *et al.* 2013: 84). This is a normal phenomenon regardless of whether or not the game’s content and form are balanced. In the case of *The Medium*, the players’ interpretation of the story told by the game does not seem to coincide exactly with the creators’ intentions, despite the fact that Marianne, as a character, is an active obstacle to bringing the player’s ideas and interpretations into the game, since, as the only character lacking a developed Spirit Self who could confront or advise her, she is an instrument of exploration of the game instead of being the subject of the player’s exploration. Piejko explains that

the team is striving to deliver something deeper too. “Playing as a medium will give you a very unique perspective that’s beyond the reach for ordinary people, and so the game’s statement is that there is no universal truth,” he explains, “there is always some grey area, and we think this topic is super-important right now when we are bombarded by media trying to shift our perspectives. “Sometimes if you crop a photo correctly it gives you a completely different message, so in *The Medium* we raise this topic, and the story is crafted so that the player will reveal more and more information which will change their perspective on what happened in the game and their opinion about the other characters.” ( Wales 2020).

Despite Piejko’s explanation concerning the lack of universal truth, it is actually difficult to identify how the gameplay is supposed to illustrate that premise. Regardless of different personal and visual perspectives included in the game, the information imparted on the players through push narrative is presented as reliable and is therefore implied to be objective—instead of, for instance, reading Thomas’ diary, the player actually re-enacts the given events as Marianne experiences them first-hand in her visions. At no point does Marianne—and, more importantly, the player—drastically change her opinion or a rigid attitude towards another character, or have to reevaluate a past event. Similarly, at no point is the player given a reason to change their opinion of Marianne, since she continues to be a reliable narrator for the entire duration of the game (in contrast to, for instance, James Sunderland in *Silent Hill 2*, whom the player supports in his search for Mary, only to find out near the end of the game that it was James himself who killed her). The interpretation of the past is clear and presents all characters as either morally justified or morally despicable, with the only truly morally debatable choice—ending Lilianne’s suffering or condemning her to a desolate, tormented life—being still easily put into perspective within either rational, responsible context, or an emotional, misguided one. In the end, Piejsko’s description of the game seems to constitute a truism more than a statement in the postmodern era.

Naturally, the control over the content and the message of the game, like any other work of art, does not belong to any single party since the meaning is made by all participants. The intention and expression of the artist(s) command neither more nor less respect than what the audience experiences. Therefore,

the element of “control” does not reside solely with the player (player choice, agency, and meaning construction) or with the game (designed experiences). Rather, control in a game play experience is an interplay of designed experiences and player projection that result in the player’s interpretation of the game’s narrative space ( Rothschild *et al.* 2013: 83).

In case of a game text the importance of content of the game (the depicted world and the characters within, as well as their fate) and the message that is supposed to be conveyed matching the form (proper tools of the game medium and appropriate formal blocks) is particularly critical. This translates into the significance of matching the atmosphere and the subject matter of the game to the most compatible genre, which would meet the player’s expectations and fittingly shape the gameplay experience. Otherwise, the resulting work might constitute a moving story, but not a moving game. *The Medium* is an example of an aesthetically remarkable work of art involving choices either illusory or abandoned. The narrative structure itself does appear to match the serious subject matter and the genre to which the game belongs; however, the lack of intervals between the subsequent quests might not only fatigue the player, but also contribute to the impression of spectatorship taking precedence over exploration and interaction. The overabundance of puzzle quests, combined with the sustained illusion of some of them constituting important parts of Marianne’s journey, translates into a situation where the player is presented with gripping content, but not the relevant toolkit that would be represented by the game mechanics.

The most obvious manifestation of the illusory nature of agency in *The Medium* is perhaps the way in which Marianne finds out that her father was also a medium; the player does indeed look for and collect the necessary clues in order to unlock the relevant information—i.e. to learn Thomas Rekowicz’s backstory—but that search is so linear and unavoidable in the context of the questline that it is, in the end, exclusively reactive in nature. The interactive aspect of that activity consists in running into those clues; this might not be an actual error of development construction, taking into consideration Bateman’s idea of following Physical Trails of Breadcrumbs ( Bateman 2021: 96-97); however, the pretense of non-linearity is negatively affected by the lack of partial rewards along the questline and the way Thomas’ backstory is presented in the scene meant to constitute the reward for the player’s efforts so far. This questline and the relevant gameplay are the part of the game in which the impression of being led by the story instead of actively uncovering it is particularly strong. Additionally, the blankness of Marianne as a main character, while not always a design failure, defines her in the primary role of being a vessel for the player to explore the story; certain design aspects as well as numerous Steam reviews show that Marianne lacks relatability, which means that she is not a factor encouraging the player to immerse themselves in the gameplay.

Due to the fact that linearity and the impression of being led by the game story are highly subjective in nature, it is helpful to refer to opinions of the players. In addition to the qualitative content, they also provide quantitative perspectives on some of the matters discussed in this article. Even limiting the data to the comments available only on *The Medium’s* Steam page, 149 out of 5,292 Steam comments taken into consideration for the purpose of this text mention linearity, or even railroading, in regards to mechanics relevant to searching and using clues. As one of the players states, the only way they could fail a given quest would be if they “ate the hints.”

*The Medium* is undoubtedly a multi-layered work of art—specifically in the sense used by Kalinowski, which would classify it as a composite artwork (cf. Kalinowski 1981: 476). Many components of *The Medium* are positioned in a structural opposition to the gameplay; this takes away player’s agency, interferes with the natural pacing of the game, and renders the story- and mechanics-related decisions ostensibly illusory and irrelevant.

It is also worth mentioning that in October 2022 Bloober Team announced the upcoming adaptation of *The Medium* as a TV series. This change of medium might prove more than beneficial for the particular story the creators of the game set out to tell, especially taking into consideration the narrative and audiovisual tools which can be employed in a TV series to create an immersive experience. *The Medium*, being a beautifully crafted—and much needed in the audiovisual discourse—story is just the work of art that might achieve a much greater success in conveying its message and refining its symbolic aspects in the TV series format than as an interactive drama.

The aim of this article was to identify and examine selected problems that emerge at the intersection of art, ambition and gameplay design. The two most prominent issues visible in many serious speculative games, which are especially conspicuous in the game that served as the primary example for this text, include the collision of the creator’s intentions with the audience experience. This is naturally not exclusive to games, but the nature of games as a medium makes this problem more jarring, and the potential negative consequences for the player experience when the game text fails to meet the contractual condition and thus the player’s expectations—more far-reaching. There are many benefits to studying those problems in more detail, but one of the most substantial findings in the context of this particular article are the observations that can realistically contribute to better, more informed decisions concerning adopting game design solutions and tailoring game content to respective game genres. Keeping those conclusions in mind can lead to games maturing and evolving as a medium—both as a part of the entertainment industry and as a mode of expression in terms of art.

## Data Availability

No data are associated with this article. The article presents a case study and relies on close reading of the secondary texts. An exception to this is the social media data from Steam. The data cannot be shared due to the ethical and copyright restrictions surrounding social media data. The Methods section contains detailed information to allow replication of the study. Any queries about the methodology should be directed to the corresponding author.

## Footnotes

^1^
The flat at the beginning of the game is a perfect example of spatial past. The game takes place in the late nineties, and the virtual environment of the apartment is full of signs and indices referring to the specific time in Polish history; however, by being so peculiar they become practically invisible on the global scale symbolism–especially for players from outside the former Soviet bloc. Cultural capital of most of the Polish players includes familiarity with the legitimate local culture depicted in the socio-historical context of the apartment as the setting of the story, e.g. the icon of Black Madonna of Częstochowa, “Solidarity” banner and pins, soviet tea glass holders, or possible association with Wisława Szymborska’s poem “Cat in an Empty Apartment.”

^2^
The cast majority of game texts include—among others—two types of scripted narrative, which are the push narrative and the pull narrative. Push narrative elements concern any information that is pushed at the player by the very game design, such as cutscenes, scripted events or voiceover. In contrast, pull narrative elements refer to the information embedded in the game world for the players to pull at, thus pulling the narrative to them (e.g. journals meant to be read) ( Calleja 2011: 123).

^3^
Whereas non-player character is a character not controlled by a player, a non-playable character is one that is not meant to be the player’s avatar, and lacks the necessary mechanics and skills of a playable character. In a very basic sense of experience design, the unique call for exploration of the gameplay (a mandatory element of the substantial majority of games) needs to be justified in mechanical elements of the gameplay as it is the particular skillset that makes a hero suited to the game exploration.

^4^
For instance, using “trail” quest structure system, which offers the player merely an illusion of non-linear choices (see van der Mer), while commonly accepted in gamebooks and visual novels, is nowadays an unpopular practice in AA and AAA size horror games. And in this case, as the Steam reviews have shown, the use of this quest structure is strongly criticized by some of the customers; also, from the narrative design perspective, some quests in *The Medium* follow the adjective paradigm (the way in which the results of players’ actions are shown) rather than the dominant verb approach, in which the players’ choices make things happen.

^5^
The Gamer motivation Model (GMM) is an empirical, study-based taxonomy of players’ motivations created by Quantic Foundry. It introduces a division of players’ interests into twelve categories, allowing to relate design patterns to user preferences and, as a result, assess the compatibility between a game and its target audience. According to GMM, player motivations are divided into three clusters that do not overlap: Action-Social, Strategy-Achievement, Immersion-Discovery. The Action-Social cluster includes the following players’ preferences: Competition, Community, Excitement, and Destruction. The Strategy-Achievement cluster includes Completion, Strategy, Challenge, and Power, while the Immersion-Discovery cluster—Discovery, Story, Design, and Fantasy. *The Medium* as a popular culture product undoubtedly fits the GMM’s Immersion-Discovery cluster of players’ preferences, but there are two observations that are worth noting. Regardless of the game including elaborate set of riddles and collection quests, *The Medium* does not reward players from the Completion subgroup of Immersion-Discovery cluster (which is quite common for the genre).

^6^
At least 72 Stream reviews, 43 negative and 29 positive ones, mention this issue as a downside of the game (observation based on reviews containing keywords: #climax #resolution, #comparedto in a way that clearly supports the given statement).

^7^
While inertia of the protagonist’s action in film texts is generally used to build suspense and allow the viewer explore a complex plot, in horror video games inertia is used to enhance the feeling of nonlinearity of gameplay. In this regard, The Medium follows rather cinematic inertia.

^8^
Keeping in mind the impressive overall Steam rating (9/10), most negative reviews focus either on the technical aspects (optimalisation, mostly) and very limited agency, or on the feeling of “watching the story rather that making it happen.” This topic is also extensively disscussed among the Steam community in the Community Discussions section. Several entries contain strong criticism, and the terms “boring” and “unplayable” recur repeatedly both in the reviews and in the comment section.

^9^
Both the sub-optimal implementation of positive discomfort and the focus on game art in the mid-game increase the ludonarrative dissonance. In words of Pamięta-Borkowska, the very essence of ludonarrative dissonance–in the context of game development–lies in the unintentional nature of its occurrence ( Pamięta-Borkowska 2019). Bloober Team might have actually knowingly allowed the emergence of this ludonarrative dissonance, but it is still worth to examine the benefit of this decision in the context of the end user experience.

^10^
The number of reviews analyzed as of the time of writing the article.
