# Supplementary material for: The problematic space between art, ambition, and gameplay: The Medium and the issues concerning difficult subject matter and gameplay in games
Source: F1000Res. 2024 Jan 5;12:784. Originally published 2023 Jul 5. [Version 2] doi: 10.12688/f1000research.133472.2 (PMC10905157; doi:10.12688/f1000research.133472.2)
Supplement: Peer review history

# Reviewer report: Reviewer response for version 2

**DOI:** 10.5256/f1000research.160465.r235890

**Published:** 2024-02-29

**Authors:** Raine Koskimaa

**Affiliations:** 1 Faculty of Humanities and Social Sciences, University of Jyvaskyla, Keskussairaalantie, Finland

The authors have made clarifications to the article, as suggested in the review. There are some changes in the main text, and some added explanations in footnotes. There is somewhat more information regarding the player review data collected from Steam. Also, the Steam materials are employed a bit more in supporting the arguments in the article.

 I do still think, however, that there is more potential in there, and the article could have been even better. As a simple thing, adding a few well selected screenshots would clarify the issues discussed considerably. The very comprehensive exposition of the game story could be shortened, and instead, the analysis of key scenes more elaborated. The way how “pacing” in the game is discussed still leaves an impression, that the authors have a very specific idea how a (horror) game should be paced, as if there was some generally acknowledge, right way to do it (and *The Medium* does not manage to live up to that standard). As a minor detail, I still don’t get the idea of the “non-playable character” – if it is not meant to be player’s avatar, then how does it differ from “non-player character”? The presentation of the Steam review data still leaves some issues open: for example, when over 40 Steam reviewers “believe that Marianne resembles a movie character rather than a typical game protagonist”, it is not fully clear, if all of those 40+ players considered that a bad thing, or if there were any reviews where the characters resembling movie characters was seen as a positive or neutral thing.

Is the case presented with sufficient detail to be useful for teaching or other practitioners?

Yes

Is the work clearly and accurately presented and does it cite the current literature?

Partly

If applicable, is the statistical analysis and its interpretation appropriate?

Not applicable

Are all the source data underlying the results available to ensure full reproducibility?

Yes

Are the conclusions drawn adequately supported by the results?

Partly

Is the background of the case’s history and progression described in sufficient detail?

Yes

Reviewer Expertise:

Game studies, temporality in digital fictions, reader-response research

I confirm that I have read this submission and believe that I have an appropriate level of expertise to confirm that it is of an acceptable scientific standard.

# Reviewer report: Reviewer response for version 1

**DOI:** 10.5256/f1000research.146464.r189933

**Published:** 2023-10-10

**Authors:** Mirosław Filiciak

**Affiliations:** 1 SWPS University, Warszawa, Poland

The article touches on the interesting and perhaps still under-represented in the literature topic of speculative video games that touch on serious issues, suggesting that this medium is particularly predestined for this. This combination, however, does not always necessarily produce a satisfying result, as is shown in a meticulous, multi-faceted analysis of the game The Medium. I admit that at times perhaps all too detailed, as the length of a text devoted to essentially one game is somewhat overwhelming. For the most part, however, this material has been brought under control, and in addition to a close reading of the game, the authors also refer to analyses from the Steam platform.

 The Authors elaborate well on the main theme, related to the illusory agency offered to players in the title under analysis. Also, reaching for reviews from the Steam platform is undoubtedly a good choice, which has allowed a very large sample to be collected (I was quite impressed to learn that these reviews were reviewed manually). A solid job has been done on the literature. What I found a little lacking in the conclusion was a broader view, moving further away from the example analysed and considering to what extent the tools and research insights developed can be applied to other games or their specific type. Despite these minor comments, my assessment of the whole is unequivocally positive.

Is the case presented with sufficient detail to be useful for teaching or other practitioners?

Yes

Is the work clearly and accurately presented and does it cite the current literature?

Yes

If applicable, is the statistical analysis and its interpretation appropriate?

Not applicable

Are all the source data underlying the results available to ensure full reproducibility?

Yes

Are the conclusions drawn adequately supported by the results?

Yes

Is the background of the case’s history and progression described in sufficient detail?

Yes

Reviewer Expertise:

Media sudies, game studies, media archaeology, cultural studies, research on media practices and infrastructure

I confirm that I have read this submission and believe that I have an appropriate level of expertise to confirm that it is of an acceptable scientific standard.

# Reviewer report: Reviewer response for version 1

**DOI:** 10.5256/f1000research.146464.r198507

**Published:** 2023-09-26

**Authors:** Raine Koskimaa

**Affiliations:** 1 Faculty of Humanities and Social Sciences, University of Jyvaskyla, Keskussairaalantie, Finland

This article has merits in how it addresses the ludo-narrative dissonance in the case of a specific game (The Medium), and how the subject matter and genre expectations play a role in there. The case the authors are making is, that implementing a complex narrative concerning serious subject matter into playable game faces fundamental problems, which are related to the expectations raised by the horror game genre where The Medium belongs. In addition to the game analysis, there is a promise of looking also at "the experiences, ideas, and interpretations the players themselves bring to the game text", and this is supposed to take place through a data set of 5000+ reviews of the game available in the Steam platform.

 The biggest problem with the draft as it is, is the imbalance between these two approaches. The game analysis is conducted in a detailed way, occupying majority of text, whereas the highly promising player review material is mentioned almost as a side thought. Furthermore, as a consequence of the game analysis emphasised, there are long passages basically just describing the game world, events and characters. These descriptions should be shortened significantly, but then, a couple of screenshots from the game (illustrating the split screen technique, for example) would be highly helpful.

 It is problematic, that the argumentation is built upon what the players expect of the game belonging to horror genre ("fails to fulfill the expectations of the player"), but then most of the discussion seems to be based upon researcher experience. Researcher play is, of course, wholly acceptable method for doing game analysis, but with strong evaluative comments, the draft tends to go close to an elaborate piece of game review.

 As there is the extensive work done with the Steam reviews, it would be highly recommendable to incorporate that material much more in the text. If it would be possible to base the argumentation explicitly on the Steam review findings, this would be a genuinely important contribution to game studies.

 Below, I provide some more detailed questions and comments:

 Throughout the text, the authors emphasises the blankness of the main character as a handicap in design. This is, however, not automatically a design failure, as there are numerous examples of a blank character employed in an effective manner. In this case there would even be an explicit motivation for that, as Marianne is a medium, channeling various things. This is one of the main issues, where examples from the Steam reviews would be necessary, to substantiate the claim.

 “The Medium as a narrative does not allow for any ambiguity in its story” – Not any ambiguity? Still you claim that “Within the narrative of The Medium, there are few moments or places of ambiguity, apart from the very ending, which unfortunately in itself makes the resolution of tension impossible.” So some ambiquity, after all?

 “She [Marianne] literally occupies the same space and time.” – this is one of the places where the temporal structure of the game is highlighted, revealing problematic complexities: what does it mean that Marianne occupies “the same space and time”? The same as…? (Later on, “Appropriately, the time and space in The Medium do not merge, but overlap, the past being always superimposed on the present”). Without deep knowledge of the game, it seems that there are two distinct ontological levels (real life / spirit world), both of them with their own temporality (even though the spirit world is focusing on the past events, it seems to have a continuum of its own). Also, in what sense do time and space “not merge but overlap”? It is probably not the necessary to engage in a deep analysis of the temporality in The Medium for the purposes of the article, but it might be worth mentioning some of the temporal characteristics, as it is a feature of the game not typical of horror games.

 “Marianne without an independent Spirit Self becomes the exception that proves the rule, making her even more unique. However, this uniqueness limits the potential for ambiguity in The Medium even further” – This is unclear. If the player cannot gain insight into Marianne's personality, doesn't this leave room for ambiguity? “In The Medium, Marianne does nothing of the sort, forcing the player to either guess or ignore her feelings and motivations. Such design strips her of subjectivity, rendering her experiences and feelings inconsequential. In practice, this means that for the player, who is in control of at least some of her actions, there is very little difference between Marianne as a character at the beginning of the game and halfway through the game.” -- How does this differ from the "iceberg" technique of Hemingway and others, limiting the narration to the events taking place, and leaving all psychologizing for the reader to conceive? Wouldn't it rather be in line of the serious subject matter not to explain everything but leaving the implications for the player to ponder upon? Isn't this a major case of ambiguity (which there is supposedly not)?

 “This strategy does not seem to be directly aimed at player’s agency, or even constitute an attempt of subverting that agency, but rather is a logical outcome of adopting a particular hierarchy of narrative tools atypical to the genre.” -- I don't understand this. What are the "narrative tools" which are "atypical to the genre"?

 “This seems to be confirmed by—among other things—the way the non-playable characters are structured, which conforms to the rules of quest design, but is used in a way more specific to gamebooks.” – Please, elaborate!

 “also due to the fact that a proper assigning of character functions in The Medium would require a more close examination of the distribution of those functions among multiple characters across different timelines (such as Spirit Thomas being a Hero in the past and the Helper in the Present) or one character being split into multiple characters” -- This seems to be in direct contradiction with the previous claim that the characters "are presented very narrowly through... one specific role".

 “Whereas non-player character is a character not controlled by a player, a non-playable character is one that is not meant to be the player’s avatar, and lacks the necessary mechanics and skills of a playable character.” – I don’t get this.

 The passage where Gamer Motivation Model is mentioned in relation to the Spirit Thomas, does not appear helpful for readers not familiar with the model (including a reference to two subgroups, which are not explained in any way). If this is considered so important that it cannot discarded, then the model should be briefly opened up here.

 “From the designers’ perspective, the player gets immersed in the story rather than their own decisions” -- What would it mean, if the case was that the players gets immersed in their own decisions?

 “Marianne’s essential function as the narrator of the game is continually reaffirmed by many elements included by game development.” -- Could you mention some examples of these elements here?

 “This further contributes to the impression that the focus of the game revolves around the game art, with history playing an important role, while playability and story—not so much.” -- Based on your own account, the story seems interesting and intricate enough.

 In terms of game design, as a result of its treatment of agency-expressing mechanics and their influence on the pacing of the story, The Medium appears to be an illustration of how not to pace a game.” -- The pacing appears in several places, but it is not clearly explained -- does "contigent on... the player's... progression" simply mean, that there is no time limit, but the player may use as much time as she wishes for solving the puzzles (in Aarseth's term, transient time)? Or the opposite (intransient time)? If so, isn't this simply stating that you don't like games with in/transient time? In any case, this reads much more like a game review than analysis.

 “at this point, the player is not so much making choices that would be rewarded with a noticeable change of the gameplay pace, as being pushed along by the plot.” -- Why would a change in the gameplay *pace* be a reward? I have a feeling, that you are using the term "pace" in some idiosyncratic way I don't get.

 “This entire sequence, however, culminates in the following exchange: [excerpt from game dialogue]” -- There are parts cut out from the exchange, which contain quite obvious intertextual reference to the opening words of Lolita by Nabokov. It is not the aim of this paper to provide a full-fledged interpretation of the game, but this kind of allusion to a well-known work providing the perspective of a childmolester might still be worth mentioning here.

 “while the game’s tense atmosphere, wide shots and particular camera angles, together with the overall aesthetics, will be readily accepted, if not welcome by the target audience, precisely due to their previous experience with other game texts of that genre, the same experience will surely render the lack of closure and confrontation as jarring and inadequate.” -- This is exactly the kind of argument, which should be supported by the large empirical data you have collected from Steam.

 “The same signs and symbols can, however, constitute an important separate dialogue within the knowledge discourse between the creative team of Bloober Team and the reviewers, specifically the Polish ones.” -- What is this referring to? Has there been some public discussion between the designers and reviewers in Poland?

 “And although it is inconsequential to discriminate between “superior” and “inferior” inertia, The Medium gameplay is paced by means of tools associated with cinematic texts rather than game texts, which means that the nature of inertia encountered in the game resembles much more the kind one would expect and appreciate in a film text.” -- What do you mean by this distinction between superior and inferior inertia? What are the tools (associated with cinematic texts) that you refer to? What kind of inertia is appreciated in film texts? Please, be more specific.

 “Unfortunately, none of the game-over scenarios constitute sufficient feedback in terms of gameplay;” -- What do you mean by "sufficient feedback in terms of gameplay" in game-over scenarios?

 “The game aesthetics (in a broad sense of the term cf. Schell (2008)) come together to create an impressive work of art at the cost of the game’s interactivity” -- This is now very thoroughly described in the previous dozen pages, and it would be interesting to hear what the players (in Steam) are saying about this obvious imbalance.

 “This scene actually constitutes the beginning of the game’s story as told by Marianne up to that point,” -- This is unclear.

 “This moment is evidently one of the few in which the creators seem to have shifted the emphasis from interactivity and agency to expression of powerful concepts and emotionally engaging experiences.” -- I am puzzled. I thought the whole argument above has been, that the designers have sacrificed interactivity for engaging storytelling throughout the game?

 “This radical artistic and design choice would fit a different audiovisual medium text, such as a film or a TV series, but falls flat in the case of a game” – Again, is there support in the Steam materials for this claim?

 “the fact that her character is instrumental, inert in terms of agency (both her own and the one she would provide to the player as an avatar), and stripped of subjectivity facilitates the creation of an atmosphere of horror and tension without increasing the stakes concerning the game progress.” -- I don't understand this, what do you mean by “increasing the stakes concerning game progress”?

 “Positive discomfort is often employed in speculative games, especially in the horror genre, but the manner in which it is implemented in The Medium is far from optimal.” -- The argumentation in this whole paragraph would need much clarification; it is not clear now, what is exactly the reason for the "less than optimal" implementation of positive discomfort in The Medium. This seems to be the key passage in this article, so it would be crucial to clarify this paragraph.

 It is a shame that the highly interesting Steam player review material is not properly presented and discussed. One would assume, that much more could be said about 700+ negative reviews, not to mention the even more interesting group of mixed reviews. It seems very strange, that such a big and important part of the research is this quickly mentioned in the article. I would suggest writing a whole chapter/section about the reviews, providing a proper qualitative analysis of it. And at the same time, the description of the game events could be much shortened, this way there could be a better balance between the game analysis and the review analysis.

 “According to Montola, the players ‘belong to a subculture of gamers that is convinced of the value of non-fun games…’” -- Which players? I assume Montola is writing about LARP players.

 “The focus on the almost theatrical take on the artistic aspect of the game at the expense of the experience of agency and gameplay, even in spite of the possibility of frustrating the players, might not necessarily be a mistake of any kind, but simply compliance with the positive negative experience trend specific to aesthetic-centered games” -- This is interesting, but would require more thorough discussion. It seems that there are two different phenomena discussed (and easily confused); the negative feelings caused by the serious, horrible subject matter, and negative feelings caused by the frustration with the design choices; these may come together in a meaningful way, but it is not clear here, if you want to say that it is the case in The Medium.

 “In the case of The Medium, the players’ interpretation of the story told by the game does not seem to coincide exactly with the creators’ intentions, despite the fact that Marianne, as a character, is an active obstacle to bringing the player’s ideas and interpretations into the game, since, as the only character lacking a developed Spirit Self who could confront or advise her, she is an instrument of exploration of the game instead of being the subject of the player’s exploration.” -- Is this based on the Steam reviews? Or purely on the researcher reading of the game?

 “At no point does Marianne—and, more importantly, the player—drastically change her opinion or a rigid attitude towards another character, or have to reevaluate a past event.” -- Earlier it is said, that there is no access to Marianne's mind in the game, so how is it possible to evaluate if she changes her opinions or not?

 “Similarly, at no point does the player change their opinion” -- Again, is the "player" here some sort of combination of the Steam reviewers, or the researcher-player?

 “Even limiting the data to the comments available only on The Medium’s Steam page, 149 out of 5,292 Steam comments taken into consideration for the purpose of this text mention linearity, or even railroading, in regards to mechanics relevant to searching and using clues. As one of the players states, the only way they could fail a given quest would be if they “ate the hints.”” -- This would be super important to look into in much more detail.

 “Many components of The Medium—or platforms—are positioned in a structural opposition to the gameplay” – What do you mean by “platforms“ here? Why is it added?

 “the game text fails to meet the contractual condition and thus the player’s expectations” -- This contractual condition would require elaboration earlier in the text

 “observations that can realistically contribute to better, more informed decisions concerning adopting game design solutions and tailoring game content to respective game genres.” -- And these observations are? It would be good to give a summary of them here in the Conclusions.

 If the results of the game analysis would be based, or supported, by the findings in the reviews, then this would be a really strong article, with original contribution.

 Concepts to define: ludonarrative, pull & push narrative

Is the case presented with sufficient detail to be useful for teaching or other practitioners?

Yes

Is the work clearly and accurately presented and does it cite the current literature?

Partly

If applicable, is the statistical analysis and its interpretation appropriate?

Not applicable

Are all the source data underlying the results available to ensure full reproducibility?

Yes

Are the conclusions drawn adequately supported by the results?

Partly

Is the background of the case’s history and progression described in sufficient detail?

Yes

Reviewer Expertise:

Game studies, temporality in digital fictions, reader-response research

I confirm that I have read this submission and believe that I have an appropriate level of expertise to confirm that it is of an acceptable scientific standard, however I have significant reservations, as outlined above.
